# Genome sequencing with gene panel-based analysis for rare inherited conditions in a publicly funded healthcare system: implications for future testing

**DOI:** 10.1038/s41431-022-01226-3

**Published:** 2022-12-06

**Authors:** Lynne J. Hocking, Claire Andrews, Christine Armstrong, Morad Ansari, David Baty, Jonathan Berg, Therese Bradley, Caroline Clark, Austin Diamond, Jill Doherty, Anne Lampe, Ruth McGowan, David J. Moore, Dawn O’Sullivan, Andrew Purvis, Javier Santoyo-Lopez, Paul Westwood, Michael Abbott, Nicola Williams, Scottish Genomes Partnership, Timothy J. Aitman, Andrew V. Biankin, Susanna L. Cooke, Wendy Inglis Humphrey, Sancha Martin, Alison Meynert, Fiona Murphy, Craig Nourse, Colin A. Semple, Nicola Williams, John Dean, Patricia Foley, Lisa Robertson, Alison Ross, Karen Williamson, Jonathan Berg, David Goudie, Catherine McWilliam, David Fitzpatrick, Elaine Fletcher, Andrew Jackson, Wayne Lam, Mary Porteous, Kate Barr, Nicola Bradshaw, Rosemarie Davidson, Carol Gardiner, Jennifer Gorrie, Rosie Hague, Mark Hamilton, Shelagh Joss, Esther Kinning, Cheryl Longman, Neil Martin, Ruth McGowan, Jenny Paterson, Daniela Pilz, Lesley Snadden, Edward Tobias, Sarah Wedderburn, Margo Whiteford, Timothy J. Aitman, Zosia Miedzybrodzka

**Affiliations:** 1 Institute of Medical Sciences, University of Aberdeen, Aberdeen, Scotland UK; 2 East of Scotland Regional Genetics Service, NHS Tayside, Ninewells Hospital, Dundee, Scotland UK; 3 North of Scotland Medical Genetic Service, NHS Grampian, Polwarth Building, Foresterhill, Aberdeen, Scotland UK; 4 South East Scotland Genetic Service, NHS Lothian, Western General Hospital, Edinburgh, Scotland UK; 5 School of Medicine, University of Dundee, Dundee, Scotland UK; 6 West of Scotland Centre for Genomic Medicine, NHS Greater Glasgow & Clyde, Queen Elizabeth University Hospital, Glasgow, Scotland UK; 7 School of Medicine, Dentistry & Nursing, University of Glasgow, Glasgow, Scotland UK; 8 Edinburgh Genomics, University of Edinburgh, Edinburgh, Scotland UK; 9 Health Economics Research Unit, University of Aberdeen, Aberdeen, Scotland UK; 10 Centre for Genomic and Experimental Medicine, Institute of Genetics and Cancer, University of Edinburgh, Edinburgh, Scotland UK; 11 North of Scotland Regional Genetic Service, NHS Grampian, Ashgrove House, Foresterhill, Aberdeen, Scotland UK; 12 Institute of Genetics & Molecular Medicine, University of Edinburgh, Edinburgh, Scotland UK; 13 Institute of Cancer Sciences, University of Glasgow, Glasgow, Scotland UK; 14 National Specialist and Screening Team, NHS National Services Scotland, Edinburgh, Scotland UK; 15 West of Scotland Centre for Genomic Medicine, Queen Elizabeth University Hospital, Glasgow, Scotland UK; 16 Genomics England, QMUL, Dawson Hall, London, EC1M 6BQ England UK; 17 University of Dundee Human Genetics Unit, Level 6, Ninewells Hospital, Dundee, DD1 9SY Scotland UK; 18 NHS Tayside Clinical Genetics Department, Human Genetics Unit, Level 6, Ninewells Hospital, Dundee, DD1 9SY Scotland UK; 19 University of Edinburgh Human Genetics Unit, Western General Hospital, Edinburgh, Scotland UK; 20 Immunology department, Queen Elizabeth University Hospital, Glasgow, Scotland UK

**Keywords:** Diagnostic markers, Genetic testing, Health policy

## Abstract

NHS genetics centres in Scotland sought to investigate the Genomics England 100,000 Genomes Project diagnostic utility to evaluate genome sequencing for in rare, inherited conditions. Four regional services recruited 999 individuals from 394 families in 200 rare phenotype categories, with negative historic genetic testing. Genome sequencing was performed at Edinburgh Genomics, and phenotype and sequence data were transferred to Genomics England for variant calling, gene-based filtering and variant prioritisation. NHS Scotland genetics laboratories performed interpretation, validation and reporting. New diagnoses were made in 23% cases – 19% in genes implicated in disease at the time of variant prioritisation, and 4% from later review of additional genes. Diagnostic yield varied considerably between phenotype categories and was minimal in cases with prior exome testing. Genome sequencing with gene panel filtering and reporting achieved improved diagnostic yield over previous historic testing but not over now routine trio-exome sequence tests. Re-interpretation of genomic data with updated gene panels modestly improved diagnostic yield at minimal cost. However, to justify the additional costs of genome *vs* exome sequencing, efficient methods for analysis of structural variation will be required and / or cost of genome analysis and storage will need to decrease.

## Introduction

Although research use of genome sequencing is now well established, evaluation of its advantages and disadvantages in the context of routine care is required to inform healthcare funding decisions.

In the UK, most healthcare, is delivered free at the point-of-care by the National Health Service (NHS), but each nation sets its own health priorities and allocates spending. In Scotland, NHS genetic testing is offered by four regional centres with clinics and laboratories. Following the dissolution of the UK Genetic Testing Network in 2018 [1], NHS Scotland labs implemented clinical exome sequencing targeted to specified disease gene bundles to replace panel tests no longer accessible from other UK genetic labs and put in place trio-based whole exome sequencing with panel-based analysis for severe developmental disorders, as the clinical utility of this test had been previously demonstrated by the Deciphering Developmental Disorders (DDD) study [2, 3].

In 2012, the 100,000 Genomes Project, was established in England to sequence 100,000 genomes from patients with cancer, rare disorders and infectious disease, and their families, in a clinical setting [4], through an NHS, academic and industrial partnership managed and implemented by Genomics England (a limited company wholly owned by the UK Department of Health). Families with rare, inherited conditions were offered genome sequencing where historic, routine genetic testing had not identified a genetic cause for their condition. Around this time, funding was made available to the Scottish Genomes Partnership (SGP), a pan-Scotland coalition of academic researchers, clinicians, clinical scientists and commissioners of Scottish healthcare, to explore the role of genome sequencing in cancer diagnostics [5, 6], rare disease cohorts [7] and population genetics [8], and also to sequence 1,000 genomes from Scottish residents with rare conditions of suspected genetic aetiology and their family members in collaboration with the 100,000 Genomes Project (SGP Study).

The primary aim of the SGP Study was to evaluate the impact of genome sequencing with collaborative analysis upon genetic diagnosis for rare disease in Scotland as well as training of clinical and scientific staff to use genomic analysis for the benefit of patients. The study was aligned with routine clinical practice: sample collection, DNA extraction, clinical interpretation and reporting of results were carried out by local clinic and lab teams. Genome sequencing was performed at a single Scottish site, with data analysis through the Genomics England panel-based informatics pipeline.

Diagnostic yield is key to the evaluation of genome sequencing in the NHS Scotland setting, and that is the focus of this paper, in the context of rare inherited disease. We also reviewed which of the additional diagnoses made by genome sequencing could have been made by the current clinical and whole exome services, which have been in place in Scotland since 2018. A detailed economic evaluation is ongoing and will be reported elsewhere.

## Subjects and methods

The SGP Study operated with regulatory approvals aligned but distinct from the 100,000 Genomes Project processes in the rest of the UK. Key differences were: (1) genome sequencing was undertaken within Scottish academic sequencing centres, and sequence data then transferred to Genomics England (i.e. no samples were sent outside Scotland); and (2) no person-identifiable data was shared outside NHS Scotland.

### Participants

Screening and recruitment ran between March 2017 and October 2018. Eligibility criteria were: meeting criteria for one of the ~200 rare phenotypes of presumed genetic origin on the Genomics England rare disease list [9], with negative test results for historic routine genetic tests specified by phenotype (e.g. microarray for intellectual disability (ID), targeted panel for a cardiomyopathy etc.). Participants reflected Scottish population ethnicity- 86.7% White British, 4.8% any other white background, 2.9% Asian or Asian British- Pakistani, 1.3% non-stated, 1.03% Chinese and 0.6% Black or Black British- African. In the ID category, in absence of malformation or prominent dysmorphism, only cases with severe or profound developmental delay were eligible. Trio-based exome analysis for developmental disorders was not available as a routine diagnostic test at the time of study recruitment. Individuals were selected at regional genomics multi-disciplinary team meetings following referral by their clinician via a standard proforma. Referring clinicians were mainly clinical geneticists but also included neurologists, rheumatologists and nephrologists. Probands were, where possible, co-recruited with parents or other family members to aid later genome sequence variant filtering by suspected mode of inheritance. Child-parent trios were preferred, though other family structures (singletons, duos, other trios, and quads) were also eligible.

Pseudonymised clinical data (phenotype under investigation, associated Human Phenotype Ontology terms, family structure) were captured in a Scotland-specific instance of Genomics England’s OpenClinica database and securely transferred to Genomics England for analysis. The cipher connecting study identifiers and person-identifiable information was retained within individual Scottish clinics for re-identification and return of results. Information on prior genetic testing (including exome analysis within the DDD study [2, 3]) was also transcribed pseudonymously from the proforma.

### DNA sampling and genome sequencing

DNA was extracted from blood, or occasionally saliva, in Regional Genetics Laboratories in Aberdeen, Dundee, Edinburgh and Glasgow using routine ISO 15189:2012 accredited processes. Minimum quality and quantity standards were 5 µg at 50 ng/µl with A260/A280 ratio ≥1.8 and no evidence of degradation, with no smearing seen when run on 0.8–1% agarose gels against a 1kB ladder, TapeStation / Bioanalyser (Agilent), Fragment Analyser (VH Bio) or Caliper GX or GX Touch (PerkinElmer).

Pseudonymised DNA aliquots were sent to Edinburgh Genomics ISO 17025:2005-accredited facility at University of Edinburgh, for short-read genome sequencing to a minimum coverage of 30X, using TruSeq PCR-free library preparation kits and HiSeq-X sequencing platform (Illumina) as described previously [7]. Each sequence data file was required to contain a minimum of 80 × 10^9^ bases with ≥Q30 from reads not duplicated or double-counted after adaptor trimming and quality trimming. Pseudonymised FASTQ files were transferred to Genomics England over a direct encrypted connection using FTP through a secure SSH tunnel.

### Clinical bioinformatic analysis

FASTQs were mapped to GRCh38 for variant calling and analysed as per the Genomics England clinical bioinformatics pipeline for genome sequencing with panel-based gene analysis [4]. This generated a list of filtered variants that were rare, co-segregated with the condition, had the correct genotype for the condition’s mode of inheritance, and were predicted to change protein structure. Variants were further prioritised into “tiers”: tier 1 predicted protein-truncating, and tier 2 predicted protein-altering variants, in genes with pre-existing evidence for involvement in the phenotype under investigation (curated within PanelApp [10] as green), and tier 3 variants were those predicted protein-truncating or protein-altering in other genes and were only analysed in trios. All other variants were untiered (e.g. common, no relevant familial segregation or non-coding) and thus not analysed. Tiered variant lists for recruited families were returned to NHS Scotland laboratories between April 2018 and August 2020 using the Genomics England Interpretation Portal and associated clinical interpretation partner platform Sapientia (Congenica Ltd, Cambridge,UK).

### Variant interpretation

NHS Scotland clinical scientists performed variant interpretation and classification of tiered variants as per ACMG [11] and UK ACGS [12] guidelines using a two-stage approach: (1) primary assessment of Tier 1 & 2 variants, for all families; and (2) secondary assessment of Tier 3 variants for those families where no confirmed genetic diagnosis was identified from primary analysis. A Scotland-wide protocol was developed to ensure consistency of secondary assessment tailored to family structure, utilising tools available via Genomics England: variants in known human disease genes with Exomiser Rank ≤5 (score 0.95) [13, 14] were reviewed for all cases; further analysis of Tier 3 variants based on alternative inheritance models (de novo, autosomal recessive and X-linked) was performed for trios and quads.

Variant review included a clinical discussion at multidisciplinary team meetings, when needed. All variant classifications were recorded by laboratories but only ACMG/ACGS Class 5 (pathogenic), Class 4 (likely pathogenic) and “hot/warm” Class 3 variants were included on diagnostic reports. Hot/warm Class 3 variants were reported since future additional clinical follow-up may yield sufficient evidence to upgrade these to a clinically-actionable variant (Class 4/5). Reported variants were validated using retained clinical DNA samples as per ISO 15189:2012 accredited laboratory processes, and formally reported back to the referring consultant clinician following standard processes. Results were returned to families as per routine practice.

Occasionally, reporting of Class 3 variants triggered additional review of clinical features or non-genetic investigations to aid re-classification of variants of unknown significance (such as biochemistry assays or radiological investigations). Clinical scientists from each service recorded classification outcomes for each family after primary and secondary assessment (and re-classification, where appropriate) using standardised templates, along with whether additional testing was required to support re-classification. Class 4 and 5 variants were considered a “confirmed genetic diagnosis”; Class 3 were categorised as “hot/warm” and “cold” variants of uncertain significance (VUS); Class 2 (unlikely pathogenic) or Class 1 (not pathogenic) variants were considered a “negative molecular diagnosis”.

Towards the end of the phase of the study reported here, targeted panel analysis of exomes for singletons with distinct phenotype bundles became available as a routine service [15] using Trusight One (Illumina) or SureSelect custom constitutional panel, alongside DDG2P-based trio whole exome analysis for severe developmental disorders (“trio-exome”) for severe and profound developmental delay and malformation. A retrospective review was undertaken by clinical scientists to indicate whether the confirmed genetic diagnoses made from genome sequencing with gene panel-based analysis would theoretically have been detected through use of these services (i.e. is the variant identified covered by the panel analysis, is the locus sequenced at sufficient depth).

### Assessment of additional diagnostic yield

Clinical report outcomes from variant interpretation were integrated with data extracted from OpenClinica (proband condition and disease category, family structure, proband age at recruitment, proband sex) and prior genetic testing in IBM SPSS Statistics 27 (International Business Machines Incorporation, worldwide). Disease categories were defined as per the Genomics England Rare Disease List [9] for the 100,000 Genomes Project. Results are shown as total numbers and percentages (with equal-tailed Jeffrey’s 95% confidence intervals) calculated using SPSS descriptive statistics and one-sample non-parametric tests.

## Results

### Proband demographics

**Fig. 1:**
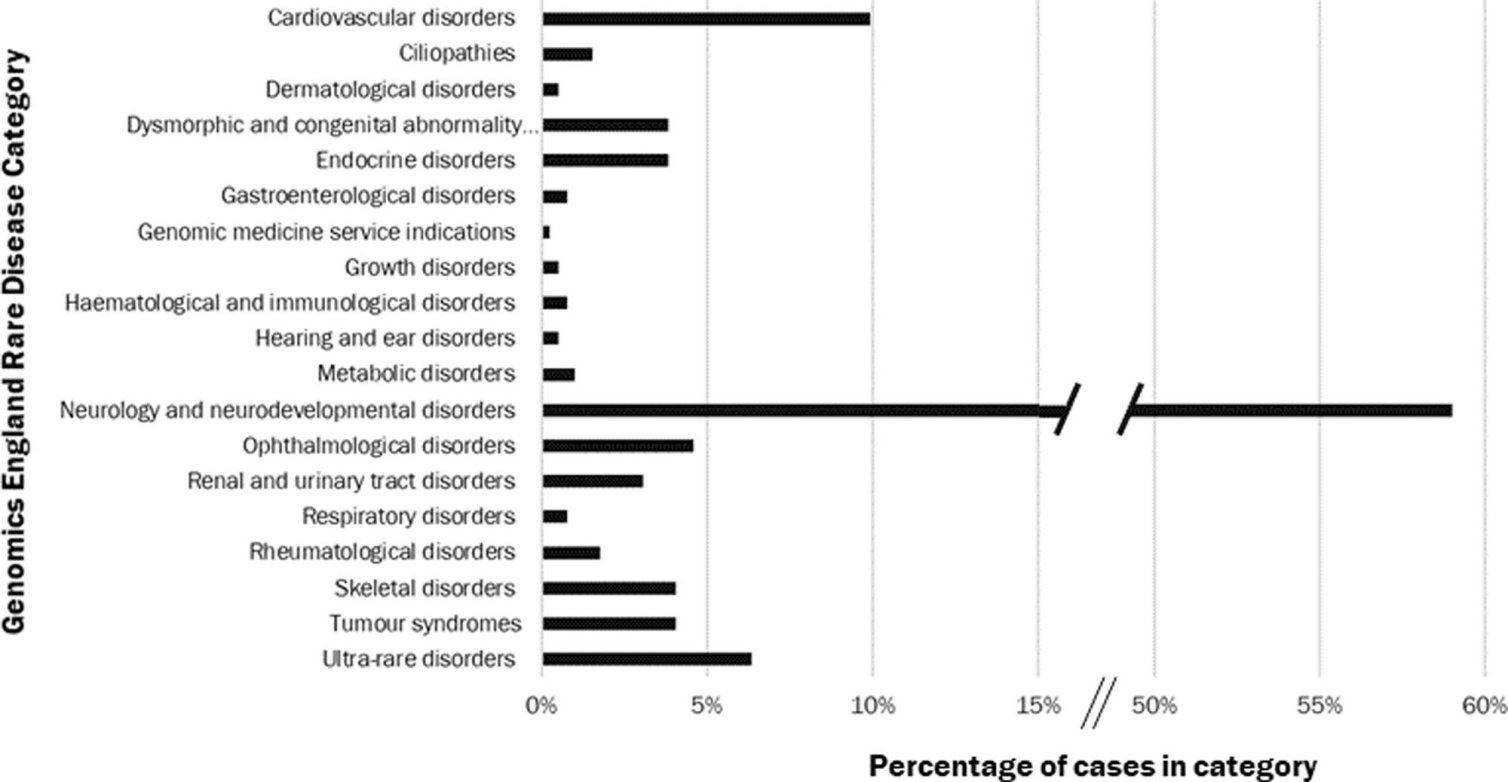
Distribution of recruitment categories among 394 probands recruited to the SGP Study. Disease categories are as those set out at Level 2 within the Genomics England Rare Disease List. Where a proband was entered with conditions in more than one category, the proband is included within counts for all relevant categories.

Of 670 eligible probands invited, 394 consented with 605 co-recruited family members (total 999). Among probands, 242 (61%) were under 16 years at recruitment and 169 (43%) were female; 258 (66%) were recruited with family members (227 (58%) parents-child trios, 13 (3%) other trios and 18 (5%) quads), with 67 (17%) singletons and 69 duos (18%). Sixty-nine relatives (11% of the co-recruited family members) had the same rare condition as the proband. Twenty-five (6%) probands had more than one condition. The majority of probands (233, 59%) were in the Neurology and Neurodevelopmental Condition category, 146 of these (63%, 37% all probands) had ID. The remainder were distributed across all other categories (Fig. 1). One family subsequently withdrew.

### Samples and sequencing

All DNA samples met the minimum sample quality standards. Genome sequence data was successfully generated for all samples, and data quality exceeded the minimum quality metrics for all samples (30X coverage and 80% bases above Q30).

### Prior testing

Probands with ID had had the following pre-testing: microarray 79%, Fragile X 20%, FISH 14 and 61% karyotype, and a mean of 3.4 panel tests (maximum 11) with diagnostic odyssey lasting 0.6 to 25 years. In non-ID, 43% had a microarray,17% a karyotype, and up to 8 small gene or panel tests (mean 2.8) and a diagnostic odyssey of 1–14.3 years.

### Variant assessment

**Fig. 2:**
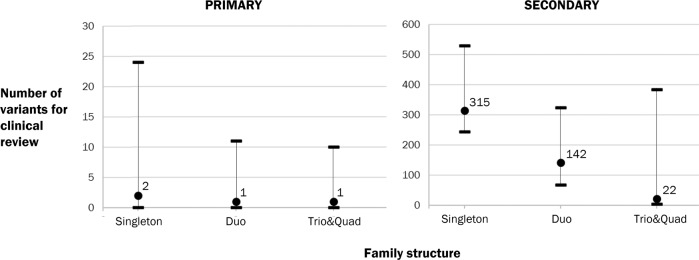
Distribution of variant numbers by family structure for primary and secondary assessment. Circles and corresponding numbers indicate median number of variants, bars and vertical lines indicate range.

One or more Tier 1 and/or Tier 2 variants were identified in 264 of 393 families (67%); all had three or more Tier 3 variants. The distribution of number of variants for Tiers 1 & 2 (primary assessment) and Tier 3 (secondary assessment) are shown in Fig. 2 for different family structures. The number of variants for secondary assessment (if required) is shown before application of filters in the protocol for secondary assessment. The number of variants for interpretation reduces with increasing family size among both Tier 1&2 and Tier 3 variants.

### Diagnostic yield - overall

**Fig. 3:**
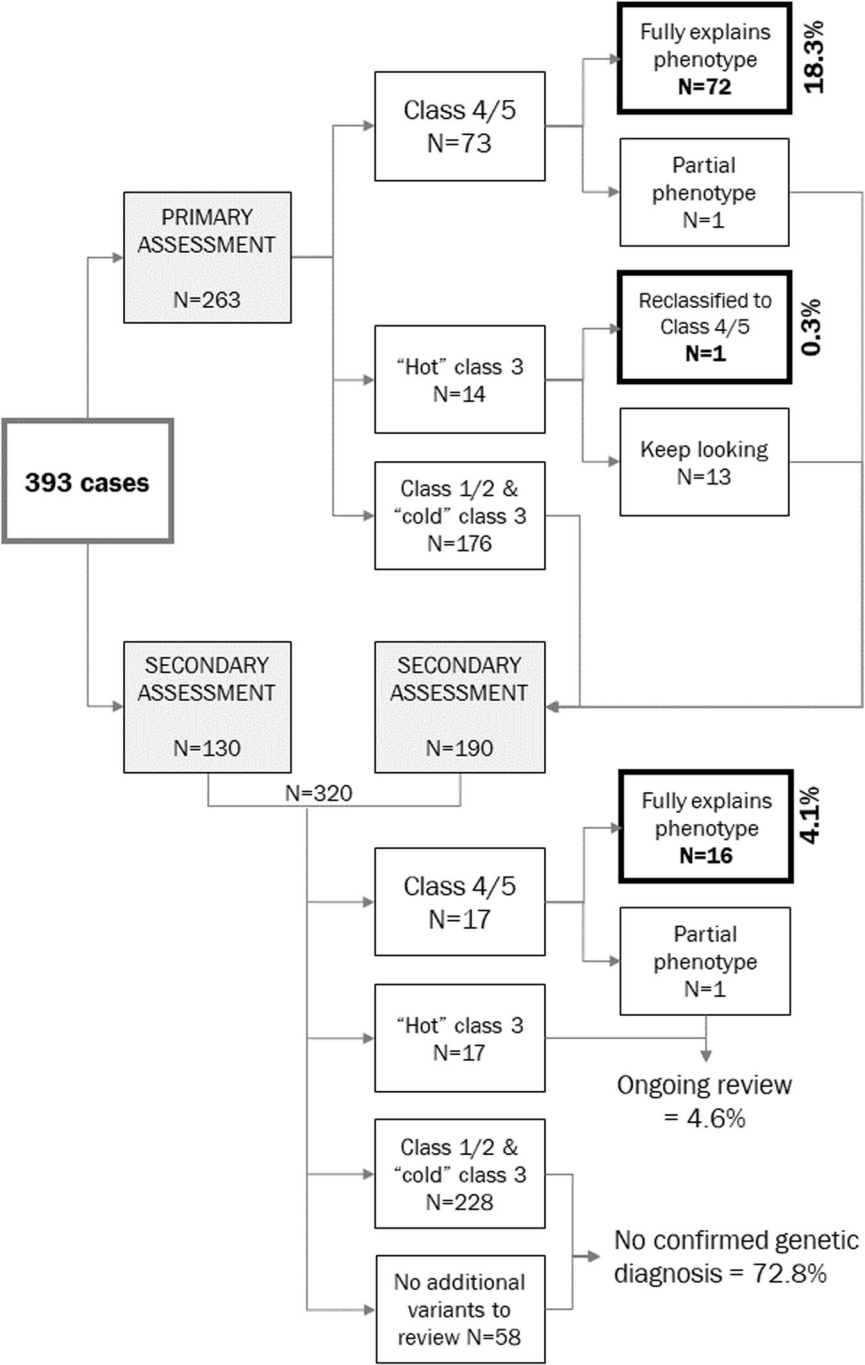
Summary of the number of cases reviewed at each stage of variant interpretation and associated outcomes. Bold-outlined boxes indicate cases where a confirmed genetic diagnosis fully explained the condition; the associated additional diagnostic yield is given alongside.

A flowchart summarising variant interpretation and associated outcomes is shown in Fig. 3. New diagnoses that fully explained the phenotype were made in 72/264 families (18.3%) following primary assessment. One further family was originally reported to have a “hot” Class 3 variant that was subsequently re-classified to a confirmed genetic diagnosis following a publication implicating the gene in disease based on this case and others (overall yield after primary assessment 18.6%). For one further family, a confirmed genetic diagnosis provided partial explanation of the phenotype - this family remains under investigation. Secondary assessment was required in 320 families, where a further 16 new diagnoses were made that fully explained the phenotype in the family (4.1%). Overall, the additional diagnostic yield for genome sequencing over and above routine testing was 22.7%, of which around one-fifth were made following secondary assessment.

Following both primary and secondary assessments, 17 families remain with “hot” Class 3 variants (4.6% families, including the one further family with partial explanation of phenotype), among which additional positive diagnoses may be identified in the future. For the remaining 286 families (72.7%), the molecular basis of the phenotype remains unidentified.

The mode of inheritance of pathogenic variants was most often autosomal dominant (AD: 60/88; 68.2%), and proven de novo in 42/50 informative trios (84%). Only three (3.4%) pathogenic variants had X-linked (XL) inheritance, and 23/88 (26.1%) were autosomal recessive (AR), with nine of these (39%) homozygous in the proband.

### Effect of family structure on diagnostic yield

“All cases” diagnostic yield varied by family structure, being highest among trios of proband plus unaffected parents (26.9%, n = 227) and lowest among duos (14.5%, n = 69). Quads and other trios had similar yields to duos (16.7%, n = 18 and 15.4%, n = 13, respectively) while yield among singletons was 19.7% (n = 66).

### Diagnostic yield by phenotype

**Fig. 4:**
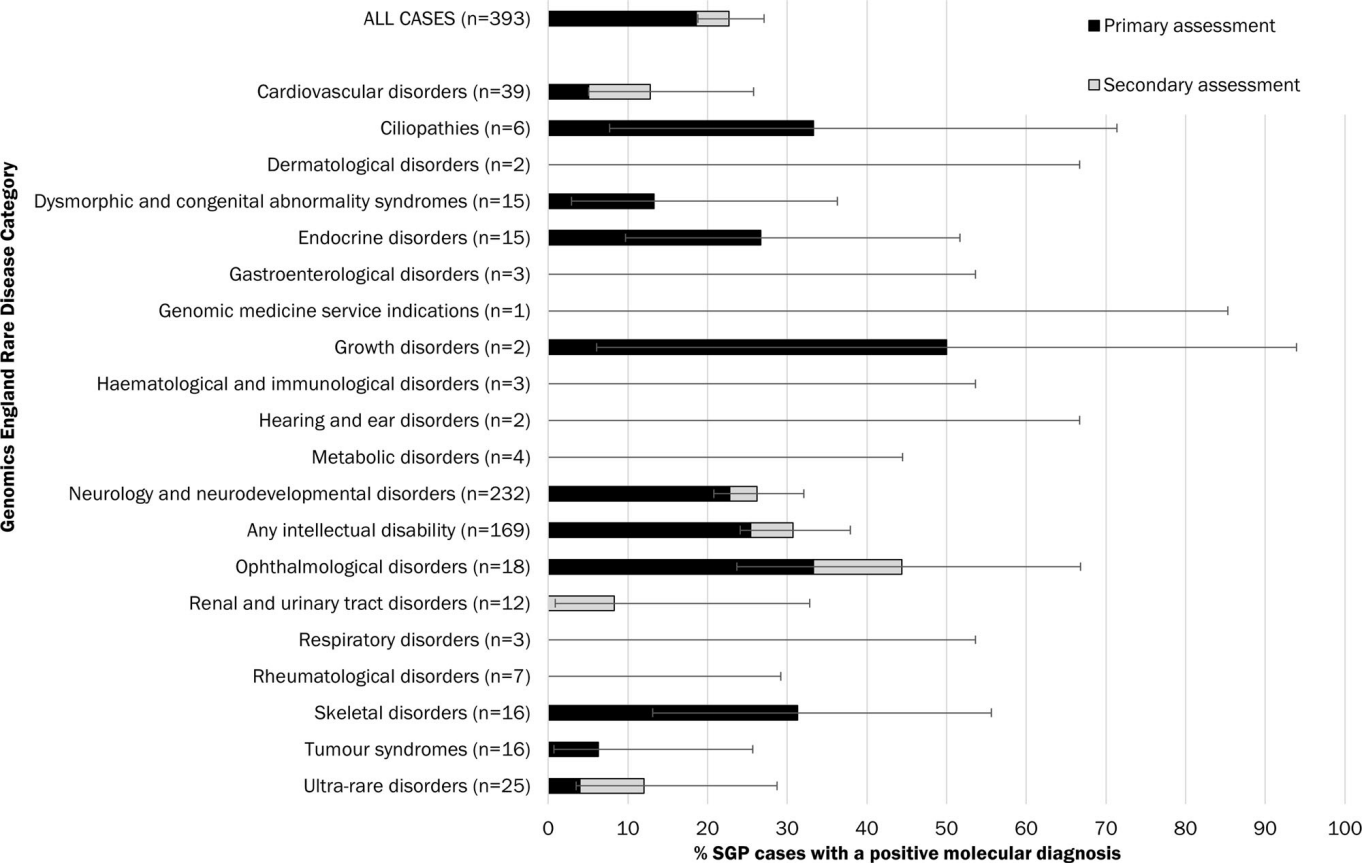
Diagnostic yield following primary (black) and secondary (grey) assessment, for all cases and by Genomics England rare disease category. Diagnostic yield is also included for probands with any ID (a subset of the Neurology and Neurodevelopmental Disorders category). The number of probands in each category is shown in brackets. Some probands are in more than one category and are included for both categories where the variant fully explained their phenotype. Bars indicate 95% confidence intervals for the overall diagnostic yield after primary and secondary assessment.

Diagnostic yield varied considerably across rare disease categories, as shown in Fig. 4. Additional details are provided in Table 1 for phenotypes with ten or more probands, where overall diagnostic yield (after both primary and secondary assessment) varied from 6.3% (Tumour Syndromes) to 44.4% (Ophthalmological disorders). Additional findings from secondary analysis were identified in Cardiovascular Disorders, Neurology and Neurodevelopmental Disorders, Ophthalmological Disorders, Renal and Urinary Tract Disorders, and Ultra-Rare Disorders.

**Table 1:** Diagnostic yield (overall) in rare disease categories with more than ten cases per group.

Genomics England rare disease category	N cases in study	Diagnostic yield [% (95% CI)]
Cardiovascular disorders	39	12.8 (5.1–25.8)
Dysmorphic and congenital abnormality syndromes	15	13.3 (2.9–36.3)
Endocrine disorders	15	26.7 (9.7–51.7)
Neurology and neurodevelopmental disorders	232	26.3 (20.9–32.2)
Ophthalmological disorders	18	44.4 (23.7–66.8)
Renal and urinary tract disorders	12	8.3 (0.9–32.8)
Skeletal disorders	16	31.3 (13.1–55.6)
Tumour syndromes	16	6.3 (0.7–25.7)
Ultra-rare disorders	25	12.0 (3.5–28.7)

For Neurology and Neurodevelopmental Disorders (n = 232), the overall diagnostic yield was 26.3%, with 41/61 (67.2%) AD, 15/61 (24.6%) AR, and 3 XL; yield was higher in ID (30.8%) versus those without (18.6%). Single cases of uniparental disomy, autosomal dominant germline mosaicism and mitochondrial inheritance were revealed in this group. A pathogenic variant was reported in 2/13 (15.4%) ID cases with epilepsy, and 2/16 (12.5%) with brain anomalies.

### Additional testing to aid variant classification

Twenty-five cases underwent additional testing, ranging from additional non-genetic sample tests (including biochemistry, histology and re-appraisal of tissue biopsy by electron microscopy and MR scans) to additional review of clinical symptoms. Of 90 confirmed genetic diagnoses achieved overall, 10 cases required additional testing to reach Class 4/5. One other VUS was lowered to Class 2. No methylation or splicing studies were required.

### Effect of prior exome testing on diagnostic yield

The effect of prior exome testing within the DDD Study was examined for participants recruited with Neurology and Neurodevelopmental Disorders.

Of 232 probands in this category, 28 were already recruited to the DDD Study. Diagnostic yield among those without prior DDD analysis was 28.9% (n = 204) compared with 7.1% among those entered in the DDD Study. A review of patient records showed that the diagnoses achieved in DDD Study participants were for two cases where the DDD Study result was not available at the time of entry into the SGP Study; and both studies identified the same result.

When limited to the subset of Neurology and Neurodevelopmental Disorders with any ID, the yield among those without prior DDD analysis (n = 122) was 36.1%, while in those with DDD analysis (n = 24), it was 4.2% (the two cases noted above).

### Detection of diagnoses by recently introduced exome tests

A retrospective review of the confirmed genetic diagnoses identified in this study indicated that 85% new diagnoses made could be achieved using the recently introduced targeted exome sequencing disease bundles or DDG2P-based trio whole exome analysis for severe developmental disorders, since the pathogenic variants detected lay within well represented regions of targeted genes. The cases where new diagnoses would not be detected were due to differences in gene panel composition between tests, typically resulting from time lags between publication of research findings and adoption onto gene panels or between updating versions of PanelApp gene panels.

## Discussion

SGP successfully performed clinical genome sequencing using the 100,000 Genomes Project data analysis pipeline for a diverse group of rare phenotype patients, obtaining new diagnoses for 23% cases where previous genetic testing had previously failed to identify a cause.

In this real world evaluation study, geographically distant Regional Genetics Services aided by colleagues from other specialties, selected and phenotyped patients to common protocols, submitted high quality DNA and sequence data to a centralised facility, and remotely accessed results from the Genomics England gene panel-based analysis pipeline to analyse, interpret, filter and report highlighted variants with minimal additional training. Clinical genomic analysis was integrated alongside routine care, providing proof of concept for use of de-identified data within the Genomics England clinical variant interpretation and data storage systems, and bringing cutting-edge clinical genomics to a geographically distributed population.

The additional yield from genome sequencing varied considerably by phenotype, ranging (among phenotypes with >9 cases) from 6.3% (95% CI 0.7–25.7%) in Tumour Syndromes to 44.4% (23.7–66.8%) in Ophthalmological Disorders. Those families who achieved a confirmed genetic diagnosis from the SGP Study after not having achieved one from historic standard testing benefitted from better understanding of prognosis and reproductive risks, giving opportunities for improved reproductive choice in future pregnancy, better ongoing care and, in a small number, the opportunity for a trial of a targeted therapy. Both the overall diagnostic yield and the variation among yield estimates from the 393 families in the SGP Study are broadly in line with those reported for the 100,000 Genomes Project pilot study in NHS England [16], and other studies of genome sequencing in rare inherited conditions [17–19]. For those phenotypes with lowest yields (Tumour Syndromes, Renal Syndromes and Cardiovascular Disorders), lower yields are likely attributable to the standard of care pre-testing comprising comprehensive targeted panels with excellent coverage; alternatively, it may be that these phenotypes are caused by complex genetic abnormalities not captured by the technology and/or identified by the analysis pipeline, such as large structural rearrangement or non-coding variation, polygenic inheritance of genes of modest attributable risk not considered within the clinical analysis pipeline, and/or phenocopies.

Yield was improved with trio analysis, being highest among proband-parent trios (yield 26.9%), and higher among singletons (19.7%) than duos or quads (14.5–16.7%), as also seen in 100,000 Genomes Pilot [16]. This perhaps suggests that yield can be maximised and cost minimised by limiting tests to family structures that are either proband plus both parents or singleton; however, our experience is that singleton analyses require far more scarce clinical scientist time, hence trios and then duos remain preferred.

Most confirmed genetic diagnoses (19%) were identified using primary assessment of filtered variants in genes implicated in the phenotype, and a further 4% from secondary assessment of filtered variants in other genes outwith selected panels. This yield is from single nucleotide variation and small indels only, since copy number variation and short tandem repeats were not returned through the Genomics England clinical analysis pipeline for all cases at the time of writing. In line with the 100,000 Genomes Project Pilot [16], it is expected that some additional yield could be achieved when these results are available, but this would require additional time of highly trained staff for analysis and validation for what may be a low number of further diagnoses.

A standard protocol for secondary assessment was used to ensure a minimum standard of review for variants of interest in genes outside the applied panels. These time-consuming secondary assessments were required in two-thirds of cases and achieved an additional 4% yield. Much of that yield arose from expansion of analysis panels following publication of new research findings. In the future, it is anticipated single-step analysis that combines primary and secondary assessment will streamline workflows. Cost-effectiveness and cost-benefit analysis of secondary assessment and regular reanalysis against the additional yield detected is required. Further secondary assessment and regular reviews are unlikely at present due to analytical staff costs, although this can be re-visited according to clinical need in individual cases if sufficiently skilled analytical time is available.

Although the genome was sequenced, variant analysis was limited to the exome by the Genomics England analysis pipeline, like other clinical pipelines for diagnosis at scale. This focussed findings to clinically reportable variants in line with current variant interpretation guidelines [11, 12]: variants in genes not proven to cause the phenotype require in vitro or in vivo evidence of a variant’s deleterious functional effect on the gene product to support Class 4 or Class 5 categorisation, which is more difficult to achieve for non-coding than coding variants and typically requires research investigation.

Twenty-eight cases that had undergone prior exome sequence analysis within the DDD study [2, 3] were included in the hope of achieving a diagnosis. Among these, two additional diagnoses were made by genome sequencing, with the same result simultaneously being identified in the DDD Study. Retrospective review of all confirmed genetic diagnoses made for cases with Neurology and Neurodevelopmental Conditions in SGP identified that these would have been found using our now routine DDG2P-based trio-whole exome sequencing pipeline for severe developmental disorders. Similarly, most confirmed genetic diagnoses made in other conditions would have been detected using targeted exome analysis current genetic testing pathways available in Scotland, supporting the assertion that genome-based sequencing with gene panel analysis offers little additional clinical utility over an exome test.

Participant data from this study is made available to the research community via Genomics England Clinical Interpretation Partnerships, and further clinically relevant results are starting to emerge for families through research analysis of data. This in turn will improve clinical gene panels as more genes are implicated in disease and evidence of functional effects in coding and non-coding variation emerges, alongside the development of reporting guidelines for non-coding variants [20].

The diagnostic yields reported here would probably all have been greater if genome sequencing was implemented as a first line test, rather than following standard of care routine testing. Where the case presentation is of a distinctive phenotype for which a panel test with good coverage is available, and the costs of genome sequencing remain of the current magnitude, targeted panel testing will likely remain the first line test of choice. However, if the presenting phenotype is complex, or if the initial panel test is negative, then trio-based genome sequencing has potential to improve the chances of a diagnosis, but this would require investment in more laboratory and clinical staff time than current standard care uses, as well as consumables and testing and data analysis infrastructure. Detailed health economic analysis of genome testing that takes a holistic view of financial and opportunity costs and benefits/value to health services and patients is ongoing to inform commissioning.

Additional diagnoses may emerge from ongoing analysis of structural variation, as new genes are implicated in disease and on further analyses for non-coding, intergenic variants, but proof of pathogenicity will remain a challenge for variants outside the exome in the short-term. Long-read sequencing is currently under way for a subset of the families in this cohort who remain without a diagnosis, to identify potentially pathogenic structural variants not identified by short read technology.

The diagnostic utility for genome sequencing with panel analysis was similar to exome sequencing in our clinical service, as suggested here and reported in published meta-analysis [18] whereas in this study and others, the cost of genome sequencing and data storage was around three times greater than that for exomes [21]. In future, cost differences may fall as sequencing and data storage improve and variant analysis becomes more automated. However, until then, and until rare variants in non-coding regions of the genome can be classified as Class 4 or 5 within the limitations of routine confirmatory testing, it will be difficult in our routine diagnostic service to justify genome analysis for most cases. However, consideration of benefits needs to focus on benefit to the patient, their family and wider societal opportunities. Workload and resource/infrastructure planning for the genetics service will also need to plan for complex variant interpretations and additional patient support needs that might be triggered by genome-based findings. This is very much a moving target as the systems/costs evolve and change and the knowledge available to support variant classification increases, and decision-making by commissioners and genetics services about which test to offer to who and when will need to be adaptable and responsive to the rapidly shifting landscape.

## Data Availability

SGP Study data are available as a subset of the 100,000 Genomes Project data via the Genomics England research environment: https://www.genomicsengland.co.uk/research/research-environment.
